# New concepts in atrial fibrillation pathophysiology

**DOI:** 10.1007/s00399-022-00897-1

**Published:** 2022-09-22

**Authors:** Ben J. M. Hermans, Vanessa Weberndörfer, Geertruida P. Bijvoet, Sevasti-Maria Chaldoupi, Dominik Linz

**Affiliations:** 1 Department of Physiology, CARIM School for Cardiovascular Diseases, Maastricht University, Maastricht University Medical Center, Maastricht, The Netherlands; 2 Department of Cardiology, Maastricht University, Maastricht University Medical Center, 6202 AZ Maastricht, The Netherlands; 3 Department of Cardiology, Radboud University Medical Centre, Nijmegen, The Netherlands; 4 Centre for Heart Rhythm Disorders, University of Adelaide and Royal Adelaide Hospital, Adelaide, Australia; 5 Department of Biomedical Sciences, Faculty of Health and Medical Sciences, University of Copenhagen, Copenhagen, Denmark

**Keywords:** Atrial fibrillation pathophysiology, Atrial cardiomyopathy, Imaging, Electrogram, Biomarkers, Pathophysiologie von Vorhofflimmern, Atriale Kardiomyopathie, Bildgebung, Elektrogramm, Biomarker

## Abstract

The current classification of atrial fibrillation (AF) is mainly focused on the clinical presentation according to the duration of AF episodes and the mode of termination, which incompletely reflect the severity and progressive nature of the underlying atrial disease. In this review article, “atrial cardiomyopathy” is discussed as a new concept in AF pathophysiology. Electrogram-, imaging-, and biomarker-derived measures and parameters to assess atrial cardiomyopathy, which will likely impact how AF is clinically classified and managed in the future, are presented.

## Abstract (de)

Die aktuelle Klassifikation von Vorhofflimmern (VHF) basiert hauptsächlich auf der klinischen Präsentation unter Berücksichtigung der Dauer einzelner VHF-Episoden und der Art, wie eine VHF-Episode spontan endet. Dies beschreibt allerdings den Schweregrad und den progressiven Charakter der zugrunde liegenden atrialen Erkrankung nicht ausreichend. Im vorliegenden Übersichtsbeitrag wird die „atriale Kardiomyopathie“ als ein neues Konzept der Pathophysiologie von VHF diskutiert. Wir stellen elektrogramm-, bildgebungs- und biomarkerbasierte Parameter für die Beurteilung der atrialen Kardiomyopathie vor, die in der Zukunft wahrscheinlich die klinische Klassifikation und Behandlung von VHF beeinflussen werden.

## Introduction

Beyond the traditional concept of reentry and the multiple wavelet hypothesis as key atrial fibrillation (AF)-sustaining mechanisms, our understanding of AF has advanced significantly in recent decades [1]. The identification of AF triggers originating from the myocardial sleeves of the pulmonary veins by Haïssaguerre and colleagues represents the basis of isolation of the pulmonary veins as the cornerstone of modern catheter ablation procedures for AF [2]. However, AF has been identified as a progressive disease (AF progression occurs in 5.5%/year) [3], which may limit the effect of rhythm control strategies in a significant proportion of patients. This progression in AF can be partially explained by different non-treated risk factors contributing to an adverse atrial remodeling process [4]. Accordingly, recent work on aggressive lifestyle and risk factor management has shown promising results in reversing AF progression and improved sinus rhythm maintenance, and the use of an integrated care approach in AF has been shown to decrease cardiovascular hospitalizations and all-cause mortality [4]. Additionally, early rhythm control strategies with combined control of comorbidities was associated with a lower risk of adverse cardiovascular outcomes than usual care among patients with early AF and cardiovascular conditions [5].

Our current classification of AF mainly focuses on the clinical presentation according to the duration of AF episodes and the mode of termination, which incompletely reflect the severity of the underlying atrial disease. In this review article, we focus on “atrial cardiomyopathy” as a new concept in AF pathophysiology and new insight in its assessment using recent advances in electrogram-, imaging- and biomarker-derived measures and parameters, which will likely impact how AF will be clinically classified and managed in the future.

## Definition of the concept

A recent expert consensus published by the European Heart Rhythm Association, Heart Rhythm Society, Asia Pacific Heart Rhythm Society, and the Sociedad Latino Americana de Estimulación Cardíacay Electrofisiología (EHRA/HRS/APHRS/SOLAECE) added “atrial cardiomyopathy” to the established pathophysiological concept of AF initiation (trigger) and perpetuation (substrate) [6]. Atrial cardiomyopathy may manifest as electrical, contractile/functional, and structural alterations. Nowadays, a combination of electrogram-, imaging-, and biomarker-derived markers are used to quantify the extent of the atrial cardiomyopathy in an individual patient. The idea is that atrial cardiomyopathy progresses: electrical manifestations are electrophysiological changes causing AF to evolve from self-limiting to more persistent AF types. Contractile/functional manifestations include increased atrial size and loss of contractile function, as well as deterioration of conduit and reservoir functions of the atria. Progression of structural manifestations is characterized by cardiomyocyte hypertrophy, atrial fibrosis, fatty infiltration, and atrial dilatation. Early stages of atrial cardiomyopathy may be largely reversible, while a progressive manifestation of the atrial disease will become more permanent and less reversible.

The concept of atrial cardiomyopathy suggests that further preventive management is still necessary despite a temporal freedom of AF.

## Quantifying atrial cardiomyopathy

Atrial structural remodeling contributes to the perpetuation of AF by conduction slowing and increased conduction heterogeneity, which has consistently been reported to occur in both experimental and clinical studies of various AF risk factors, including hypertension, obesity, obstructive sleep apnea, diabetes mellitus, metabolic syndrome, heart failure, valvular heart disease, and endurance training [7]. Atrial fibrosis contributes to AF persistence through discontinuous conduction that favors reentry or preferential conduction as well as anchoring of AF drivers [4, 7]. Electrogram-, imaging-, and biomarker-derived markers to quantify atrial cardiomyopathy are summarized in Table 1.

**Table 1:** Measures to quantify atrial cardiomyopathy (ACM)

Measurement method	Parameter
Electrogram-derived	
Surface ECG	Prolonged P wave, P‑wave terminal force in ECG lead V1
Electrocardiographic imaging (ECGi)	Reduced conduction velocity, prolonged activation time
Electroanatomical mapping	Low voltage areas, prolonged activation time, reduced conduction velocity
Imaging-derived	
Echocardiography	Dilated atrium, atrial emptying function, atrial strain derived by speckle-tracking
Magnetic resonance imaging (MRI)	Dilated atrium, impaired atrial function, atrial sphericity, fibrosis (late gadolinium enhancement)
Computer tomography (CT)	Dilated atrium, impaired function, atrial sphericity, fatty infiltration, left atrial appendage shape
Biomarkers-derived	
Blood	Natriuretic peptides (→ atrial dilatation/stretch) Fibroblast growth factor (→ fibrosis) C-reactive protein, interleukin 6 (→ inflammation) Factor VIII, von Willeband Factor (→ hypercoagulability)
Genetics	Genome-wide association studies derived common genetic AF susceptibility variants used in polygenic risk scores
Digital	Signals (e.g., photoplethysmography, single lead ECG) derived from digital devices

### Electrogram-based atrial substrate mapping

**Fig. 1:**
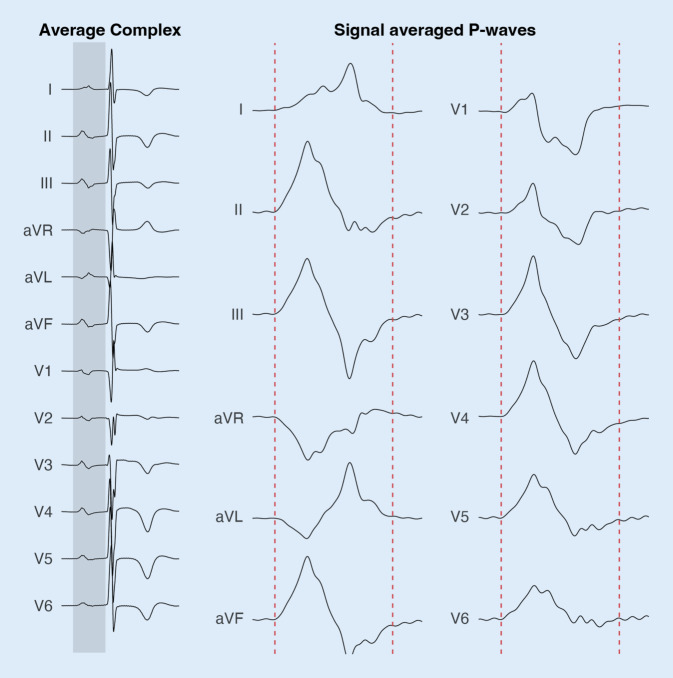
Electrocardiogram (ECG)-derived measures to quantify atrial cardiomyopathy: average ECG complex (*left*) and signal averaged P‑wave (*right*)

In addition to the diagnosis of AF in a 12-lead resting electrocardiogram (ECG), signal processing approaches on ECGs (Fig. 1) are increasingly used to characterize atrial conduction properties, which have been shown to have some predictive value for ablation outcome and AF recurrences after cardioversion. Artificial intelligence-enabled ECG algorithms can detect AF with a high accuracy using 12-lead or single-lead ECGs and could even identify the presence of AF from a single ECG recorded during sinus rhythm. Although most of these artificial intelligence models have not yet reached clinical routine, they could have an important implication in AF screening [8].

Electrocardiographic imaging (ECGi) is a new technique to study conduction properties by non-invasively estimating the conduction on the heart surface from a body-surface potential map and the patient’s heart and torso anatomy [9]. ECGi has mostly been used to study ventricular arrhythmias, but is increasingly also studied in AF.

During invasive clinical electrophysiological ablation studies, the current standard mapping approach involves three-dimensional (3D) electroanatomic voltage mapping to identify areas of low voltage or regions with increased electrogram fractionation [7]. Low-voltage areas (typically < 0.5 mV during sinus rhythm) in the atrium have been associated with endocardial scar and/or structural defects, although the threshold can vary with rhythm change. Several multielectrode mapping catheters are currently in use for detailed high density electroanatomic mapping. These catheters have 16–64 electrodes and different interelectrode spacing.

### Imaging-based atrial substrate mapping

**Fig. 2:**
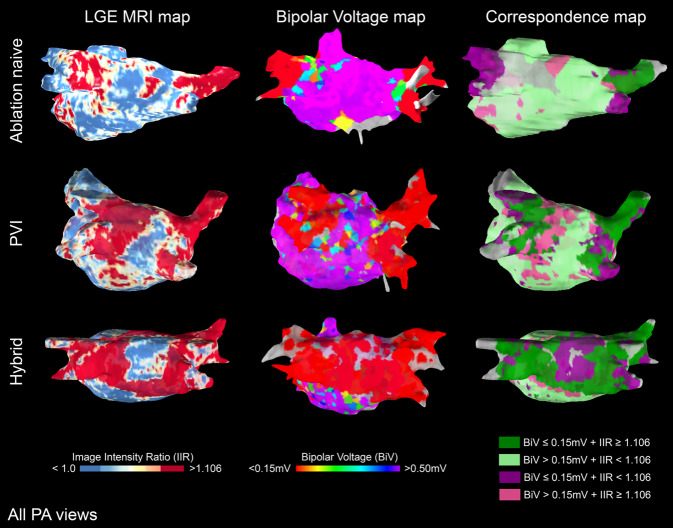
Assessment of atrial structural substrate and atrial fibrillation ablation scars: late gadolinium enhancement (*LGE*) magnetic resonance imaging (*MRI*) maps (*left*) and bipolar electroanatomical voltage maps (*right*) of a patient who was ablation-naïve (*top*), a patient who had undergone intracardiac pulmonary vein isolation (*PVI*) (radiofrequency catheter ablation), and a patient who had undergone epicardial hybrid atrial fibrillation ablation (radiofrequency ablation clamp). In both technologies, *red* represents scar regions. (For color codes of the image intensity ratio and bipolar voltages, see below the figure; all posteroanterior views)

Advances in late gadolinium-enhanced magnetic resonance imaging (LGE-MRI) allow for noninvasive assessment of structural alterations in atrial cardiomyopathy. In LGE-MRI, a gadolinium-based contrast agent accumulates in areas of increased extracellular space. However, it is currently not possible to differentiate between LGE areas caused by replacement fibrosis, necrosis, inflammation, edema, or interference with adjacent hyperenhanced structures, such as epicardial fat. Additionally, different LGE-MRI postprocessing methods lead to discrepancies regarding extent and regional distribution of atrial cardiomyopathy, as well as the correlation to low-voltage assessed in endocardial voltage mapping [10]. The contrasting results may be related to technical challenges with LGE-MRI, including spatial resolution, motion artifact, irregular heart rates, and the quantitation of LGE, which can be algorithm-dependent. In a recent randomized controlled trial, LGE-MRI-guided fibrosis ablation plus pulmonary vein isolation, compared with pulmonary vein isolation catheter ablation only, resulted in no significant difference in atrial arrhythmia recurrence [11]. In addition to the assessment of atrial remodeling, the evaluation of ablation scar has also been approached by LGE-MRI (Fig. 2). A better integration of LGE-MRI information into the AF ablation pathway (e.g., interventional cardiac MRI) may result in better outcomes in the future [12].

**Fig. 3:**
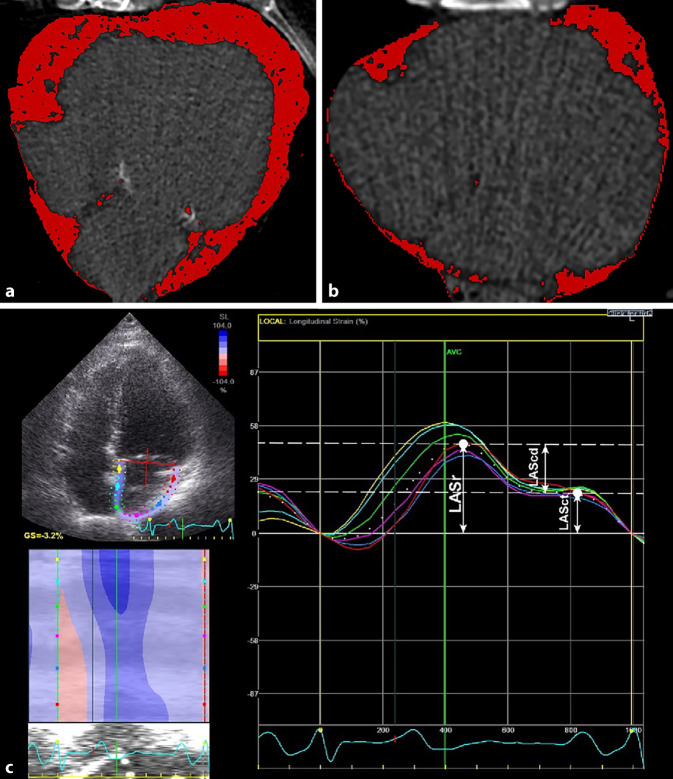
Assessment of epicardial adipose tissue volume by computer tomography (**a**, **b**). Patient A has a total volume of 476 ml and patient B 73 ml epicardial adipose tissue (*red*). Assessment of functional impairment of atrial deformation properties by speckle-tracking echocardiography (*left upper panel*) with focus on left atrial reservoir (*LASr*), conduit (*LAScd*) and contractile (*LASct*) strain (*left lower and the right panel*) (**c**)

In addition to fibrosis and ablation scar, epicardial adipose tissue, which has been shown to contribute to atrial remodeling processes and AF progression by its paracrine effects, leading to increased fibrosis or direct fatty infiltration in the contiguous atrial myocardium, can also be quantified by computed tomography (Fig. 3a, b) or MRI [13]. Epicardial adipose tissue volume has been associated with increased risk of developing AF, AF persistence, and postablation recurrences independent of other measures of adiposity [14]. Interestingly, electroanatomic mapping has confirmed more pronounced changes with larger low-voltage areas in the posterior and inferior left atrium that are adjacent to the posteriorly located increased epicardial adipose tissue seen on MRI [14]. These findings may support a targeted ablation strategy, such as posterior left atrial isolation, and the early initiation of lifestyle and pharmacological interventions to reduce bodyweight.

In addition to structural alternations, functional impairment of atrial deformation properties also represents an important component of the progressive atrial remodelling and AF substrate. During ventricular systole, left atrial strain derived by speckle-tracking echocardiography reflects left atrial expansibility and stiffness (Fig. 3c). In patients with paroxysmal AF, left atrial strain is related to individual and combined comorbidities [15].

### Biomarkers and genetic architecture

Indications for atrial cardiomyopathy could potentially be detected in community settings based on quantification of circulating cardiovascular biomolecules (Table 1) or digital biomarkers derived from wearable devices (e.g., photoplethysmography, ECG, and heart sound signals [16]). Genome-wide association studies have also identified many common genetic AF susceptibility variants throughout the genome. Polygenic risk scores derived from these can be used to successfully predict a person’s risk of developing AF [17].

## Summary and future perspectives

Atrial cardiomyopathy can explain the nature of AF (stable vs. progressive vs. regressive) in individual patients. Emerging diagnostic strategies integrating electrogram-, imaging- and biomarker-derived measures and parameters will help to further objectively quantify atrial cardiomyopathy and guide its management. This may result in a more personalized assessment and targeted treatment of atrial cardiomyopathy to modify the substrate responsible for AF perpetuation. Meanwhile, electrophysiologists must integrate aggressive lifestyle and risk factor management as one of the main pillars of AF care, given the established evidence of its role in delaying or even reversing atrial remodeling and improving AF outcomes.

## Declarations

For this article no studies with human participants or animals were performed by any of the authors. All studies mentioned were in accordance with the ethical standards indicated in each case.
